# A New Whole Genome Culture-Independent Diagnostic Test (WG-CIDT) for Rapid Detection of *Salmonella* in Lettuce

**DOI:** 10.3389/fmicb.2020.00602

**Published:** 2020-04-17

**Authors:** Dele Ogunremi, Andrée Ann Dupras, Sohail Naushad, Ruimin Gao, Marc-Olivier Duceppe, Katayoun Omidi, Imelda Galván Márquez, Hongsheng Huang, Lawrence Goodridge, Roger C. Lévesque, Nur A. Hasan, Manoj Dadlani, Brent Dixon, Sebastian Magierowski, Luke Masson

**Affiliations:** ^1^Ottawa Laboratory Fallowfield, Canadian Food Inspection Agency, Ottawa, ON, Canada; ^2^Department of Food Science and Agricultural Chemistry, McGill University, Sainte-Anne-de-Bellevue, QC, Canada; ^3^Institut de Biologie Intégrative et des Systèmes, Université Laval, Quebec City, QC, Canada; ^4^CosmosID Inc., Rockville, MD, United States; ^5^Bureau of Microbial Hazards, Food Directorate, Health Canada, Ottawa, ON, Canada; ^6^Department of Electrical Engineering and Computer Science, York University, Toronto, ON, Canada; ^7^National Research Council of Canada, Montreal, QC, Canada

**Keywords:** culture-independent, metagenomics, lettuce, *Salmonella*, whole genome, bioinformatics

## Abstract

The rapid detection of foodborne microbial pathogens contaminating fresh fruits and vegetables during the intervening period between harvest and consumption could revolutionize microbial quality assurance of food usually consumed raw and those with a limited shelf life. We have developed a sensitive, shotgun whole genome sequencing protocol capable of detecting as few as 1 colony forming unit (cfu) of *Salmonella enterica* serovar Typhimurium spiked on 25 g of lettuce. The Ion Torrent sequencing platform was used to generate reads of globally amplified DNA from microbes recovered from the surface of lettuce followed by bioinformatic analyses of the nucleotide sequences to detect the presence of *Salmonella*. The test is rapid and sensitive, and appropriate for testing perishable foods, and those consumed raw, for *Salmonella* contamination. The test has the potential to be universally applicable to any microbial contaminant on lettuce as long as a suitable bioinformatics pipeline is available and validated. A universal test is expected to pave the way for preventive and precision food safety and the re-shaping of the entire spectrum of food safety investigations from the current disease-limiting, reactive procedure to a proactive, disease prevention process.

## Introduction

Consumption of fresh fruits and vegetables is increasingly linked with outbreaks of foodborne illnesses, and the bacterial pathogen *Salmonella* is one of the most commonly incriminated causes (Centers for Disease Control and Prevention, 2005; Lynch et al., 2009; Kozak et al., 2013). Human foodborne *Salmonella* infection is consistently one of the most burdensome of all microbial food safety hazards due to a combination of high disease prevalence and relatively high rates of hospitalization and deaths of exposed people (Batz et al., 2011; Thomas et al., 2015). Prevalence of foodborne salmonellosis has remained high throughout the world, with an estimated global mortality rate of 155,000 individuals due to non-typhoidal salmonellosis (95% Confidence Interval: 39,000 – 303,000 deaths; Majowicz et al., 2010). Over 2,600 serovars of *Salmonella* have been identified and, while only a restricted number of these serovars are commonly associated with foodborne illnesses (Guibourdenche et al., 2010; Jackson et al., 2013), the diversity within and among these serovars is considerable and can limit effective detection, isolation and recovery of *Salmonella* organisms from food. For example, selective enrichment procedures critical for the laboratory isolation of *Salmonella* from contaminated food, which include the use of tetrathionate-based media and/or Rappaport-Vassiliadis broth, differentially favor the growth of different *Salmonella* organisms and can result in different recovery outcomes (Kumar et al., 2010; Lee et al., 2017). In food matrices contaminated with low numbers of *Salmonella*, recovery of organisms may be very difficult or unsuccessful and this could explain the discrepancy between reports of low prevalence of *Salmonella* in food surveys and high prevalence of clinical salmonellosis in humans. Hydrogen sulfide production by *Salmonella* is one of the most relied upon attributes for culture diagnostics because of the characteristic black colonies growing on commonly used agar plate such as XLT-4, however non-H_2_S producing *Salmonella* are increasingly identified in food animals (Jurinović et al., 2018) and humans (Albert et al., 2014). Worse still, non-pathogenic enteric bacteria such as *Proteus* and *Citrobacter* are capable of mimicking *Salmonella* leading to false positive results (Tate et al., 1990; Eigner et al., 2001; Park et al., 2012). While the use of non-optimal culture procedures could contribute to this dismal picture, other testing procedures currently used for the control of foodborne salmonellosis require improvement to achieve a significant reduction in foodborne salmonellosis. Recall of contaminated food is a widely used tool to forestall distribution and consumption of potentially contaminated or implicated food. As part of the epidemiological investigation into clinical cases of foodborne illnesses, interviews are conducted on patients to identify food consumed in the days leading to the clinical episode. Information provided by the patient on food consumed could conceivably lead to a narrowing of food to be targeted for testing, however the answers provided may not be reliable for two main reasons. The incubation period for foodborne illnesses could vary from a few hours to as long as 3 weeks, or even longer in the case of *Listeria monocytogenes* (Public Health Agency of Canada^1^), and patients may not readily remember the type of food consumed in the days prior to experiencing symptoms. Apart from difficulties in remembering what was consumed, a second reason limiting the identification of contaminated food as a source of a foodborne illness is that a patient may not have been aware of all the constituents of a meal and may not be able to accurately or comprehensively answer the question on what type of food was consumed. Thus, the source of contamination is not often identified in many *Salmonella* outbreaks and this may explain why the prevalence of foodborne salmonellosis has not decreased appreciably despite a high volume of testing and on-going regulatory surveillance for the presence of the pathogen in food products. Studies aimed at developing a rapid and sensitive method for detecting contaminants in fresh produce should reduce the overall prevalence of foodborne salmonellosis, which has remained high over the last decade (Public Health Agency of Canada, 2017), and lead to improved food safety.

As a foodborne pathogen, *Salmonella* displays a number of attributes which makes it a suitable model foodborne contaminant for evaluating a new approach to food safety. Its high prevalence, relatively high degree of hospitalization accompanied by some degree of mortality, when combined with the frequency of food recall should make it possible to accurately assess the impact of implementing a new, disruptive test aimed at transforming current food safety procedures. The life cycle of *Salmonella* involves both extracellular and intracellular phases and by mirroring what may be expected of a purely extracellular foodborne pathogen such as *Escherichia coli* or for an intracellular organism such as *Listeria monocytogenes*, the results obtained for *Salmonella* can be loosely extended to other organisms for the purpose of general improvement in food safety practices.

An effective testing procedure should at the minimum detect doses of a contaminant that could cause an illness upon ingestion. The number of *Salmonella* organisms required to cause illness in people is estimated at approximately 100 colony forming units (cfu) (McCullough and Eisele, 1951; Vought and Tatini, 1998; Hara-Kudo and Takatori, 2011), and for this reason a practical test will need to have adequate analytical sensitivity, targeting a lower range of contamination ideally at 1 cfu per serving.

This study was undertaken to develop a new, sensitive and robust shotgun whole genome sequencing, culture-independent diagnostic test (WG-CIDT) that can be applied universally to identify a bacterial contaminant in food and which can be completed over the time period between harvesting and consumption of fresh vegetables.

## Materials and Methods

### Bacteria Culture

*Salmonella enterica* serovar Typhimurium strain 22495, an isolate for which the genome sequence has been determined (Ogunremi et al., 2017a), was retrieved from our inventory of bacterial glycerol stock, inoculated into Brain-Heart infusion (BHI) broth and incubated at 37°C overnight. To prepare the bacterial contaminant for the spiking experiment, overnight culture was diluted 1:10 and incubated for 1 h at 37°C. The cell density was adjusted to 10^8^ cfu/ml (OD^600^∼ 0.30) in preparation for contaminating the surface of lettuce.

### Lettuce Preparation and Processing

Outer leaves of heads of romaine lettuce (*Lactuca sativa* L. var. *longifolia*) obtained from local retail stores were pre-screened for the presence of *Salmonella* using a published multiplex PCR procedure (Ogunremi et al., 2017b) before use. Individual lettuce leaves (approximately 25 g) were placed in a humidified plastic container and incubated at 37°C with shaking at 80 rpm for 3.5 h, after which the leaves were placed in a stomacher bag containing 40 ml of peptone water supplemented with 0.1% Tween. The stomacher (Seward, Worthing, England) was used to dissociate microbes from the surface of the lettuce under a carefully calibrated condition of 115 rpm for 1 min, chosen to minimize the disruption of plant cells and to also limit the release of plant DNA. Fluid recovered from each bag was passed through a filter paper (Whatman No 1; GE Healthcare Life Sciences, Mississauga, ON, Canada) followed by syringe filtering with a 5 mm disk (Versapor membrane; Pall Corporation)^2^ fitted to a 60 ml syringe to remove any plant material. The bacteria present in the filtrate were pelleted by centrifugation at 17,500 × *g* for 60 min (Sorvall RC 5B, DuPont Company, Wilmington, DE, United States) and the pelleted lettuce surface extract was used for downstream PCR analysis. For the spiking experiment, a 25 g lettuce leaf was placed in a humidified plastic container and spiked with an estimated 1-10^6^ cfu of *S.* Typhimurium followed by incubation at 37°C with shaking at 80 rpm leading to the generation of pelleted lettuce surface extract as described above. Non-spiked lettuce was included in each experiment and tested in parallel. The exact count of *Salmonella* delivered to the surface of lettuce was determined by plating a replicate aliquot of the bacterial suspension used for the spiking experiment on Brain Heart infusion (BHI) and Xylosine Lysine Tergitol-4 (XLT-4) agar plates followed by an overnight incubation at 37°C and plate examination and colony enumeration the following day.

### Multiplex PCR for *Salmonella*

A multiplex PCR procedure was used to pre-screen lettuce obtained from retail stores and to monitor the presence of *Salmonella* bands following intentional contamination. The procedure was based on the use of four primer pairs designed to amplify DNA fragments or a portion of genes of *Salmonella enterica* namely, *invA* (211 bp), *iroB* (309 bp) and *STM4497* (523 bp) and a control bacterial ribosomal DNA (1,026 bp) as previously described (Ogunremi et al., 2017b). The PCR cycling conditions consisted of an initial denaturation step at 94°C for 5 min, and 35 cycles of denaturation (94°C for 1 min), annealing (62°C for 2 min) and extension (72°C for 2 min). A final extension step at 72°C for 10 min was included. Samples were analyzed by electrophoresis on 1.5% agarose gel with a voltage of 100 V for 90 min. Bands were visualized (Gel Doc EZ Imager, Bio-Rad Laboratories, Mississauga, ON, Canada) on an agarose gel after staining with ethidium bromide over 15 min, followed by a rinse with distilled water for 5 min. Lettuce samples shown to contain *Salmonella* bands during the PCR pre-screening procedure were deemed contaminated from source and excluded from further experiments.

### DNA Extraction

When purified DNA obtained from the surface of lettuce was required as the starting material for global DNA amplification, the pellet of the lettuce surface extract was re-suspended in 100 μl of distilled water and DNA extracted using the Wizard^®^ Genomic DNA Purification Kit (Promega, Madison, WI, United States). Purified DNA was assessed for quality by absorbance reading at optical density values of 260 and 280 nm (OD_260__/__280_) using a spectrophotometer (DU 730 Beckman Coulter, Mississauga, ON, Canada), and was quantified by using a fluorometer (Qubit 2.0, Life Technologies, Carlsbad, CA, United States).

### Global DNA Amplification

Three whole genome linear amplification kits namely, GenomePlex (Sigma Aldrich, Oakville, ON, Canada), REPLI-g (Qiagen, Hilden, Germany) and GenomiPhi (GE Healthcare, Mississauga, ON, Canada) were assessed to determine an optimal procedure for ensuring that sufficient starting DNA material from the pelleted bacterial extract was available to detect the presence of *Salmonella* on spiked lettuce. For the GenomePlex kit, pelleted lettuce surface extract was suspended in 9 μl of distilled water followed by the addition of 1 μl of buffered proteinase K lysis solution (2 μl Proteinase K solution + 32 μl of lysis buffer) and incubation at 50°C for 1 h and proteinase K inactivation at 99°C for 4 min. The DNA solution was stabilized ahead of library preparation and the DNA amplification step by adding 2 μl of library preparation buffer and 1 μl of library stabilization buffer followed by incubation in a thermal cycler at 95°C for 2 min. With the tube placed on ice, 1 μl of library preparation enzyme was added followed by cycling at 16, 24, and 37°C each for 20 min, and a final 5 min incubation at 75°C. DNA amplification of the stabilized library was carried out by adding an amplification master mix (10X; 7.5 μl) and DNA polymerase (5 μl) and distilled water (to make up to 75 μl, i.e., 48.5μl). The amplification temperature conditions were 95°C for 3 min (initial denaturation), and 25 cycles of 94°C for 30 s (secondary denaturation), and 65°C for 5 min (annealing and extension). For the REPLI-g kit, denatured DNA was prepared from harvested bacteria by adding a suspension of the bacteria in distilled water (4 μl) and mixing with buffer D2 (3 μl; DTT and DLB buffer) and heating at 65°C for 10 min, followed by the addition of a stop solution (3 μl) to terminate the reaction. Denatured DNA (10 μl) was added to a mastermix solution (10 μl) containing REPLI-g DNA polymerase (2 μl) and linear amplification carried out at 30°C for 8 h. At the end of the amplification, DNA polymerase was inactivated at 65°C for 3 min. For the GenomiPhi V2 kit, purified DNA was extracted and quantified as described above and added (1 μl) to sample buffer (9 μl) and mixed with a mastermix containing random hexamer primers (10 μl) and heated at 95°C for 3 min followed by a cooling step to allow the primers to anneal. DNA amplification was accomplished by the addition of the Phi29 DNA polymerase and nucleotides followed by incubation at 30°C. A final step involved the inactivation of the polymerase at 65°C for 10 min. Amplified DNA samples prepared with these kits were stored overnight at 4°C before use or at –20°C for longer periods. Assessment of the performance of each kit in achieving optimal amplification, as required to facilitate the detection of *Salmonella* sequences in spiked samples, was based on the presence of *Salmonella* bands in agarose gel analysis of the amplicons generated after carrying out the *Salmonella* multiplex PCR procedure (Ogunremi et al., 2017b).

### Library Preparation and Whole Genome Sequencing

Globally amplified DNA (100 ng) prepared from pelleted lettuce surface extract was used to prepare each DNA library made up of 400 bp fragments for the purpose of whole genome sequencing on the Personal Genome Machine (PGM) or Ion S5 Torrent sequencing platform. The DNA libraries were prepared using Ion Express DNA Library kit (Thermo Fisher, Burlington, ON, Canada) according to the manufacturer’s instructions by following a series of steps, namely: enzymatic fragmentation, adaptor ligation and size selection of globally amplified DNA (Pippin Prep, Beverly, MA, United States). Libraries were amplified and the concentrations and size distribution of DNA fragments in each amplified library was assessed by means of an Agilent Bioanalyzer using the High Sensitivity kit (Agilent, Santa Clara, CA, United States). For the PGM, sequencing templates were prepared by carrying out emulsion PCR on the Ion One-Touch ES^TM^ equipment using the Ion PGM^TM^ Hi-Q^TM^ View OT2 Kit (Thermo Fisher). The resulting ion sphere particles were enriched for templates, diluted as necessary, before loading on the Ion Torrent 318 chip with the aid of the Ion One Touch 2 (Life Technologies) preparatory to sequencing on the PGM over 7 h^3^. For Ion S5 sequencing, template preparation and Ion 530 chip loading were carried out using an automated procedure (Ion Chef; Thermo Fisher). Given the high capacity of the 530 chip, two libraries were loaded per chip and contents of two 530 chips were sequenced in each run over 4.5 h. At the end of the PGM and S5 runs, the fastq file for each sample was downloaded from the Ion Torrent server for bioinformatic analysis.

### Bioinformatics Analysis: Detection of *Salmonella* Genome Markers, Assembly, MEGAN Analysis and CosmosID Analysis

Four bioinformatics approaches were used to detect *Salmonella* sequences present in the raw reads obtained following the Ion Torrent metagenome sequencing of spiked lettuce surface, namely (a) presence of the *invA* gene of *Salmonella* spp., (b) presence of a 523-bp DNA fragment specific for *S.* Typhimurium, (c) MEGAN analysis of *de novo* assembled contigs and (d) k-mer based taxonomic analysis using CosmosID bioinformatics software. For approaches (a) and (b), fastq files containing raw reads were imported into CLC Genomics software (Qiagen, version 6) and analyzed for the presence of the *invA* gene, which is a well-known marker specific for *Salmonella* (Galan and Curtiss, 1989) or of the nucleotide sequence of the previously described *S.* Typhimurium-specific 523-bp DNA fragment (Ogunremi et al., 2017b). The *invA* gene sequence (1,996 bp) and the Typhimurium-specific 523-bp DNA fragment were excised from assembled *S.* Typhimurium strain 22495 (Gen Bank accession No CP017617) and were used as the template upon which to map raw reads sequences. For MEGAN analysis, *de novo* genome assembly of the trimmed fastq reads was performed with the using the MEGA-HIT assembler (Li et al., 2015) and exported as fasta files followed by identification of the contigs by BLAST analysis based on 99% identity^4^. The fasta files were downloaded in XML format and imported into the MetaGenome Analyzer, MEGAN version 6.0 (Huson and Mitra, 2012)^5^. The result is the taxonomic analysis of the organisms recovered from the lettuce and speciation of the microbial contaminant inferred from the contigs assembled from DNA fragments generated from extracts from the surface of the lettuce. For approach (d), unassembled sequence reads were directly analyzed by CosmosID metagenomic software as described elsewhere (Meisel et al., 2018; Yan et al., 2019) to reveal taxonomic composition of the community. Briefly, the system utilizes a high performance data-mining k-mer algorithm and highly curated dynamic comparator databases (GenBook^®^) that rapidly disambiguate millions of short reads into the discrete genomes or genes engendering the particular sequences. The cloud-hosted web portal can be accessed at: https://app.cosmosid.com/login. The pipeline has two separable comparators: the first consists of a pre-computation phase for reference database and a per-sample computation. The input to the pre-computation phase is a reference microbial genome or antibiotic resistance and virulence gene database, and its output is phylogeny trees, together with sets of variable length k-mer fingerprints (biomarkers) that are uniquely identified with distinct nodes, braches and leaves of the tree. The second per-sample, computational phase searches the hundreds of millions of short sequence reads or contigs from draft assembly against the fingerprint sets. Overall classification precision is maintained through aggregation statistics. Enhanced detection specificity is achieved by running the comparators in sequence. The first comparator finds reads in which there is an exact match with a k-mer uniquely identified with a reference genome or antibiotic resistance or virulence gene; the second comparator then statistically scores the entire read against the reference to verify that the read is indeed uniquely identified with that reference. For each sample the reads from a species are assigned to the strain with the highest aggregation statistics. Sequence raw reads in fastq file format uploaded to the CosmosID webportal^6^ and a comprehensive result of the closest organisms consequently matched; prophage sequences and antimicrobial resistance patterns were generated within a few minutes, typically less than 5 min. The detection threshold based on the relative abundance of matching biomarkers was set at 0.02% based on our optimized protocol. Except for the genus-specific *invA* gene, the other three procedures infer not only the identification of *Salmonella* but also the characterization of the contaminant at the serovar level, i.e., Typhimurium.

## Results

### Use of Multiplex PCR to Evaluate Optimal Conditions for Detecting *Salmonella* Targets Recovered From Spiked Lettuce

**FIGURE 1:**
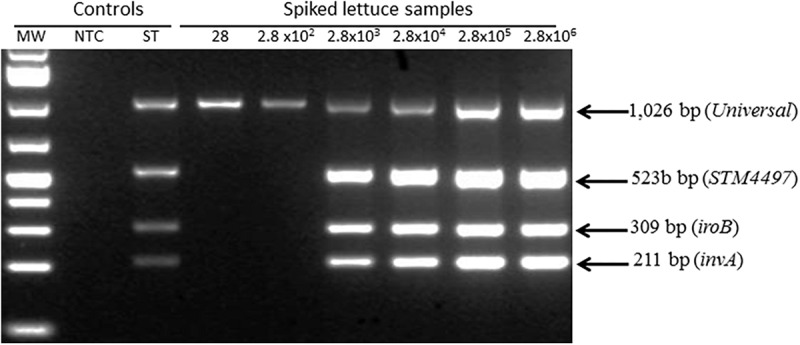
Detection of *Salmonella* contamination on spiked lettuce using multiplex polymerase chain reaction. Lettuce was spiked with *Salmonella* between 28 – 2.8 × 10^6^ colony forming units per 25 g sample. Material from the surface was immediately extracted and analyzed for the presence of three *Salmonella* fragments derived from *invA*, *iroB* and a Typhimurium-specific STM4497 gene. *Salmonella* count was determined by titration plating on Brain Heart Infusion agar to estimate the numbers applied to each lettuce sample.

Toward the goal of developing a rapid and robust WG-CIDT, we investigated and established conditions under which *Salmonella* DNA sequences present in a sample of lettuce (25 g) spiked with a low number of the bacterial cells can be reproducibly detected. We tested for the minimum contamination dose that can be detected by a *Salmonella* multiplex PCR test performed on extracts prepared from the surface of lettuce immediately after intentional contamination and without any further analysis. We detected samples of lettuce contaminated with over 10^3^ *Salmonella* bacteria but not samples exposed to 10^3^ bacteria or fewer (Figure 1).

### Evaluation of Global DNA Amplification Procedures as Assessed by a Multiplex PCR Detection of *Salmonella* Fragments

**FIGURE 2:**
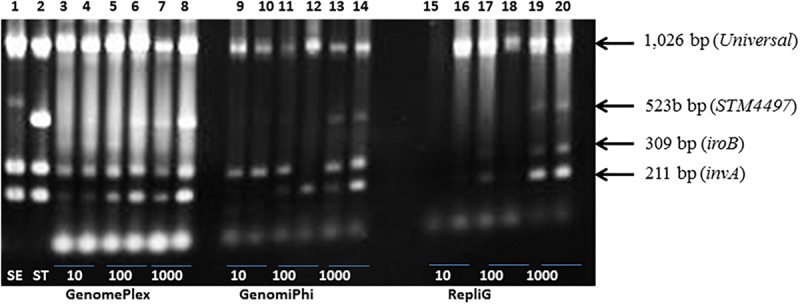
Comparison of the performances of three whole genome amplification procedures for the PCR detection of *Salmonella* fragments on the surface of spiked lettuce. Three genome amplification kits were analyzed namely: GenomePlex (Lanes 3–8), GenomiPhi (Lanes 9–14) and RepliG (Lane 15–20). Lettuce samples (25 g) were spiked with 10, 100, and 1000 colony forming units of *Salmonella* Typhimurium in duplicates. Positive controls consisting of pure cultures of *Salmonella* Enteritidis, SE (Lane 1) and *Salmonella* Typhimurium, ST (Lane 2).

We investigated whether a global amplification of the entire DNA composition of the extract will lead to improved, specific detection of *Salmonella* contamination by multiplex PCR. A secondary objective was to identify the kit that will facilitate rapid detection of *Salmonella* using the fastest protocol. Three amplification kits were evaluated, namely GenomiPhi, REPLI-g and GenomePlex. PCR analysis of globally amplified DNA from lettuce (25 g) that was spiked with <10 cfu of *Salmonella* resulted in the detection of at least 2 of the 3 *Salmonella* PCR targets (*invA*, *iroB*) using any of the global amplification procedures (Figure 2 and Supplementary Table 1). Except for the very low (<10 cfu/25 g of lettuce) to low numbers of exposure (e.g., up to 30 cfu/25 g of lettuce), all three procedures resulted in reliable amplification of the *Salmonella* targets (Figure 2). At very low numbers, the GenomePlex procedure showed a superior performance in that the *invA* and *iroB* targets were readily detected. When contaminated with low to medium numbers (10^1^ – 10^3^ cfu/25 g of lettuce), all three targets were readily detectable. For examples, all three targets were detectable in lettuce contaminated with 17 (3 samples), 140 (3 samples) or 146 cfu (3 samples) or higher. The only exceptions were replicate samples exposed to 27 cfu in which the 523-bp DNA fragment was not detected. In the same manner, the GenomiPhi kit detected all three bands when lettuce was inoculated with high numbers of bacteria (2,050 cfu; *n* = 2 samples) but inconsistently when exposed to 1–27 cfu range (*n* = 10 samples). All three targets were detected in the majority of samples inoculated with medium doses, but not all. Inoculation with 140 cfu and higher (7 samples) resulted in the detection of all targets, but only one target was detected in one sample with similar exposure level (180 cfu), and two targets only in two other samples (400 cfu). The REPLI-g kit was also sensitive in amplifying targets of ≥10 cfu/25 g of lettuce. Lettuce with very low numbers of *Salmonella*,≤10 cfu/25 g, were inconsistently detected. All samples spiked with <10 cfu (*n* = 4 samples) were detected, although the amplification of the fragments was inconsistent, ranging from 1 – 3 fragments. All three *Salmonella* fragments were readily detected in lettuce containing > 10 cfu when analyzed with the REPLI-g kit however, in a few cases (17-27 cfu; *n* = 3), only the two smaller fragments were detectable. The time required to complete the global amplification step varied from one kit to another and mainly depended on the number and duration of the incubation steps, as follows: GenomePlex (∼4 h), REPLI-g (∼8 h) and Genome Phi (∼16 h). Because of its sensitivity in detecting low numbers of contaminants, consistent performance and short incubation period (Figure 2) with acceptable DNA fragment lengths (data not shown), the GenomePlex kit was used in subsequent global amplification procedures to develop the shotgun metagenomic sequencing protocol.

**FIGURE 3:**
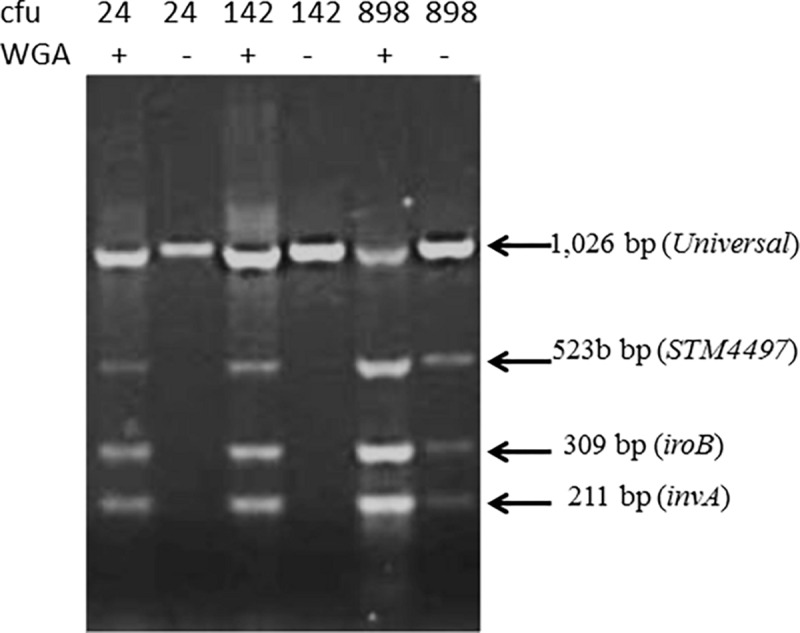
Sensitivity of the detection of *Salmonella* from surface extract of lettuce (25 g) exposed to varying numbers of colony forming unit (cfu) incubated for 3.5 h before extracting surface contaminants. One set of samples was subjected to whole genome amplification (WGA). Genome amplification was necessary to detect *Salmonella* DNA fragment in lettuce exposed to low numbers (i.e., 24 and 142 cfu).

To further improve the sensitivity, without unduly prolonging the procedure, we incubated intentionally contaminated lettuce for 3.5 h before testing, extracting contaminants and carrying out whole genome amplification with the GenomPlex kit, and found a more consistent performance especially with the lowest numbers of organisms (Figure 3).

### Direct Sequencing Analysis of Lettuce Surface (Spiked and Non-spiked)

**FIGURE 4:**
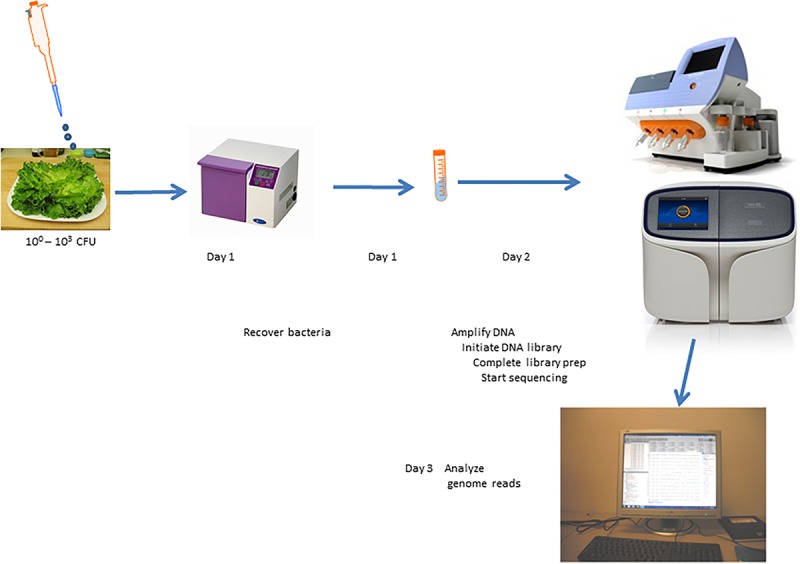
Scheme for culture-independent testing of *Salmonella* contamination of lettuce using Ion Torrent genome sequencing platform.

**FIGURE 5:**
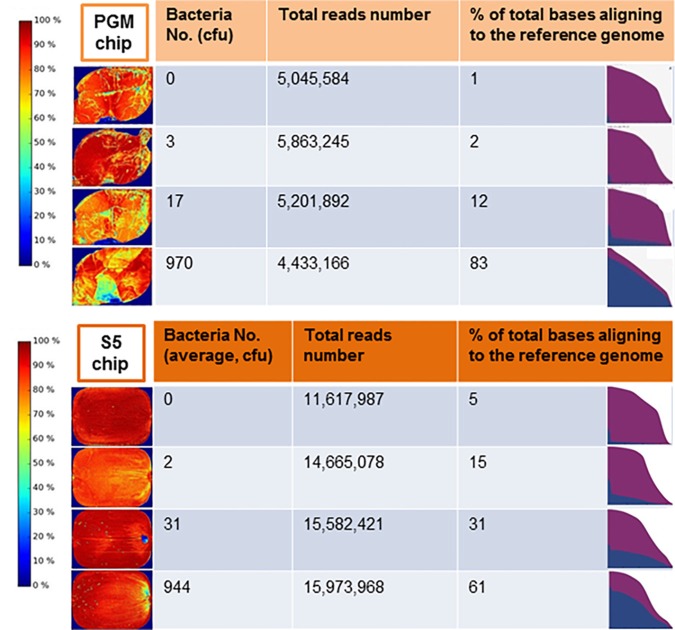
Display of the Ion Torrent **(A)** Personal Genome Machine (PGM) server after the completion of sequencing runs for an non-spiked lettuce sample (0 colony forming unit, cfu) compared to samples contaminated with 3, 17, or 970 cfu of *salmonella* Typhimurium and **(B)** S5 server for non-spiked lettuce sample compared to samples contaminated with average of 2, 31, or 944 cfu of *Salmonella* Typhimurium. The heat map (left panel) indicated the density of DNA on the surface of the sequencing chip. The area under the curve in the graph (right panel) showed the abundance of *Salmonella* sequences (blue) relative to non-*Salmonella* reads. A quantitative estimate of bases from raw reads aligned to the refence genome is shown as %.

Comprehensive genome sequencing of globally amplified DNA from spiked and non-spiked lettuce was achieved using two Ion Torrent sequencing instruments, namely the PGM and Ion S5 (Figure 4). The two sequencers use the same general chemistry and the same sequencing principle, and display the results soon after the sequencing run, including the degree of alignment of the reads with a known reference genome that has been chosen for this purpose. In this case, *S.* Typhimurium strain 22495 genome provided a quick assessment of sample contamination with *Salmonella* (Figure 5). In addition, the downstream data analyses between the two sequencers were identical, however the S5 is designed to generate more reads per run. In summary, 0.3–6.2 million (M) reads (average = 4.1 M) were generated using the PGM for each sample or metagenome in contrast to 1.5–18.2 M reads (average = 7.6 M) for each of two samples loaded on each chip of the Ion S5 sequencer. Under the conditions described in this study, the Ion S5 can be used to test four metagenomes per run given that two chips can be loaded per machine run.

### Bioinformatic Analysis of Shotgun Metagenomic Sequencing Reads From Non-spiked Lettuce

**FIGURE 6:**
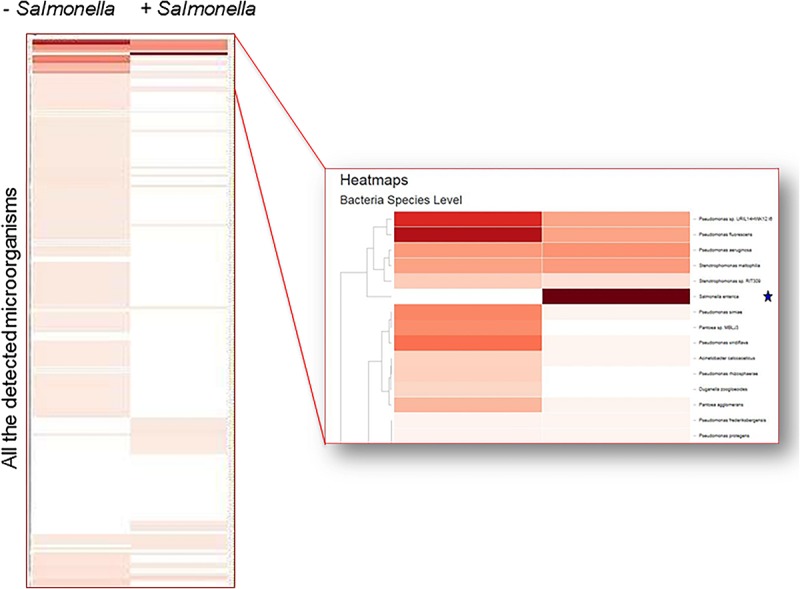
Metagenomic detection of contaminants on the surface of lettuce. Heat map shows the detection of *Salmonella* in spiked sample (*) but not in a non-spiked control.

A large number of bacterial genera were found on the surface of lettuce as determined by CosmosID analysis using k-mers. The 16 most prominent bacterial genera consistently found on the surface of fresh romaine lettuce were: *Pseudomonas, Pantoea, Acinetobacter, Flavobacterium, Methylobacterium, Sphingomonas, Pedobacter, Chryseobacterium, Xanthomonas, Bacillus, Achromobacter, Pseudoxanthomonas, Enterobacter, Pandoraea, Dyella*, and *Citrobacter* (Figure 6). Comparable results were obtained when MEGAN analysis, the only other bioinformatic approach that generated phylogenetic trees, was performed on the same samples (data not shown).

### Bioinformatic Analysis of Shotgun Metagenomic Sequencing Raw Reads From *Salmonella-*Spiked Lettuce Samples Generated Using the Ion Torrent PGM

The raw sequence reads obtained following Ion Torrent metagenome sequencing of spiked lettuce were analyzed to detect the presence of *Salmonella* sequences by means of four bioinformatics approaches, namely (a) detection of sequences matching the *invA* gene of *Salmonella* spp., (b) detection of sequences matching the 523-bp DNA fragment specific for *S.* Typhimurium, (c) MEGAN analysis of *de novo* assembled contigs and (d) k-mer analysis based on proprietary CosmosID analysis.

All four bioinformatics procedures led to the consistent detection of *Salmonella* sequences generated with the Ion Torrent PGM from all the spiked lettuce samples from which ≥10 cfu were recovered from replicate samples (*n* = 15 samples; 100% sensitivity).

Among the contaminated lettuce samples (*n* = 10) from which 1 – 9 cfu of *Salmonella* were recovered from their replicate samples, one sample tested negative for *Salmonella* sequences when analyzed by each of the four bioinformatics procedure (Table 1). Two of the remaining samples tested positive by CosmosID analysis which was able to characterize both isolates to the serovar level (i.e., *S.* Typhimurium) and with MEGAN analysis which was only capable of detecting them to the species level (i.e., *S enterica*). Detailed comparison of the data from each analysis showed that the CosmosID procedure was the most sensitive in detecting and characterizing lettuce spiked with very low numbers (9 out of 10 samples) whereas MEGAN analysis was also very sensitive identifying *Salmonella* contaminants in the same nine samples at the species level and most of them (7 out of 10) at the serovar level. The *invA* gene marker detected a majority of the samples (6 out of 10 samples) whereas the 523-bp DNA fragment detection was the least sensitive (3 out of 10 samples) for the samples contaminated with very low numbers of *Salmonella*. The automated analysis on the Ion Torrent PGM server based on alignment on sequenced nucleotides against a preloaded reference genome of *S.* Typhimurium detected samples spiked with low, moderate or high numbers of *Salmonella* organisms with very high sensitivity (Figure 5). We expected that the sensitivity of the CIDT would diminish if the number of reads were sub-optimal, and were, therefore, surprised that a sample from which only 0.34 M reads were generated tested positive using all four bioinformatic procedures. This sample was, however, contaminated with 100 cfu which represents a moderate level of contamination. In the same vein, all four bioinformatic methods detected another sample contaminated with only 10 cfu but with over twice as many reads (0.75 M). All in all, only one out of 10 samples contaminated with very low numbers (<10 cfu) tested negative when sequenced with the PGM.

**TABLE 1:** Culture-independent detection *of Salmonella* sequences in spiked lettuce.

	**Spiking level**	***Salmonella* organisms (cfu/25g lettuce)**	**No of samples**	**No of reads (Millions)**			**Detection (No of positives)**				**%**
				**Min**	**Max**	**Average**	**invA**	**523bp**	**CosmosID**	**MEGAN**	**Detected**
	Nil	0	8	2.84	6.17	4.39	0	0	0	0	0
	Very Low	1, 1, 2, 3, 4, 5, 5, 7, 8, 9	10	0.75	6.12	4.47	6	3	9	9	90
PGM	Low	10, 16, 17, 20	4	1.44	5.2	4.06	4	4	4	4	100
	Medium	100, 140, 140, 140, 320 970, 970, 970	8	0.34	6.14	3.39	8	8	8	8	100
	High	13400, 132317, 1321488	3	1.37	5,86	4.09	3	3	3	3	100

	Nil	0	6	2.44	16.68	8.72	0	0	0	0	0
	Very low	1, 1, 2, 3, 4, 5, 5, 7	8	1.49	10.17	6.47	7	4	8	8	100
S5	Low	10, 16, 16, 17, 46, 70, 88	7	2.07	18.22	8.47	7	7	6	6	100
	Medium	117, 180, 182, 571, 680, 810	6	3.13	8.49	6.21	6	6	6	6	100
	High	1800, 2880, 11240 26080 226989	5	6.34	9.81	8.03	5	5	5	5	100

*The presence *of Salmonella* in spiked and non-spiked samples was determined by analyzing metagenome sequence raw reads developed using DNA obtained from the surface of lettuce samples and analyzed by four bioinformatic procedures based on the detection of *invA* gene or a 523 bp DNA fragment specific for *Salmonella* Typhimurium, or with the aid of CosmosID proprietary software or an open source MEGAN software. cfu, colony forming unit.*

### Bioinformatic Analysis of Shotgun Metagenomic Sequencing Raw Reads From *Salmonella*-Spiked Lettuce Samples Generated Using the S5 Sequencer and Compared With the Output From the PGM Sequencer

Apart from using the S5 sequencer to confirm the detection of lettuce spiked with low to high numbers of *Salmonella*, we were particularly interested in whether a higher number of reads will translate to consistent detection of lettuce spiked with very low numbers of *Salmonella* (<10 cfu). Specifically, we wanted to know if the single sample that tested negative after PGM sequencing could be detected with a deeper sequence coverage. In addition, we wanted to compare the performance of the different bioinformatic procedures to detect *Salmonella* present in low doses at higher sequence coverage. To optimize the use of the higher capacity S5 sequencer, we generated the sequences of two barcoded samples per sequencing run using the 530 chip; the average number of reads for the S5 was approximately 1.9 times higher than that of the PGM per sample (4.1 M for PGM versus 7.6 M for S5). All samples exposed to very low numbers of *Salmonella* were detectable by CosmosID and MEGAN bioinformatic procedures when sequenced with the S5 sequencer at a higher coverage than the PGM sequencer. In particular, the PGM-negative sample (5.2 M reads) was S5-positive (9.7 M reads) with CosmosID and MEGAN analyses. The detection of both *InvA* and 523-bp DNA fragment showed marginal increases with the higher S5 coverage (average = 7.6 M reads). Comparison of the bioinformatic procedures on data generated with the S5 sequencer showed that the CosmosID and MEGAN analysis identified the *Salmonella* sequences in all the spiked samples. Relative abundance level of *S.* Typhimurium sequences using CosmosID in very low dose contaminated samples was ≥0.04% (*n* = 8 samples; sensitivity = 100%. In addition, background readings was negligible at 0–0.02% indicating a high specificity with negligible spurious detection of *Salmonella* sequences in non-spiked, negative samples. MEGAN analysis was also 100% accurate and identified *S.* Typhimurium sequences at the serovar level. The *invA* gene sequences was detected in 7 out of 8 samples. The *invA*-negative sample (1 cfu) had a run size of 4.13 M reads with the S5 which was below average and was similar to what could be expected with a PGM sequencing (Table 1).

## Discussion

We have developed a culture-independent, direct sequencing procedure that allows a very sensitive and rapid detection of *Salmonella* on lettuce. The procedure led to the detection of all spiked samples containing 1 or more cfu per 25 g of lettuce once an adequate number of sequence reads, estimated at ≥6 M reads per sample using an Ion Torrent sequencer, was available for bioinformatic analyses. Success in developing the test and in attaining a high level of sensitivity was contingent on rigorous experimentation and careful analyses regarding specific factors, namely, (a) ensuring that an adequate number of DNA templates of the contaminant are generated and (b) comparing different bioinformatics approaches for detecting sequences specific for the pathogen. These factors will now be discussed in greater detail. Sample preparation procedures were carried out to minimize unwanted DNA, i.e., plant DNA, which can be expected to dominate a typical DNA extraction procedure of surface contaminants of lettuce. Thus, we took deliberate and rigorous steps to reduce cells of lettuce origin and, thereby proportionally increase the amount of microbial contaminants in the extract from the surface of lettuce. Our procedure involved a very gentle dislodging of microbes present on the lettuce surface which was experimentally determined by evaluating different settings of the stomacher and choosing one that avoided significant damage to plant cells. Whenever plant cells were significantly damaged, possibly from aged lettuce, the sample turned green because of the contaminating chlorophyll. Thus, we ensured that samples were barely visibly green before proceeding to the next step. Furthermore, particulates that may have broken off from the leaves were filtered off using a 5 μm sieve that will allow bacteria to pass through the pores but retain larger albeit non-visible plant particles prior to the bacterial lysis step. The choice of the sieve size allowed for minimal loss of bacterial cells. These procedures apparently translated to a more sensitive detection of *Salmonella* DNA from intentionally contaminated lettuce.

Reducing plant source material was carried out simultaneously with increasing the amount of pathogen source DNA by an innovative enrichment procedure given that a selective increase may not be attainable. The many steps involved in generating genome sequences from a contaminated food sample, starting from the initial handling of the sample to preparing sequencing templates, raises the possibility that even a small loss in the material of interest at each step could cumulatively result in a very low signal, thereby reducing overall test sensitivity. To ensure that there was enough *Salmonella* DNA present at the time of sequencing of material from lettuce surface extract, we carried out two non-selective “enrichment” procedures which demonstrably contributed to the excellent sensitivity observed. The first procedure was an incubation step of the contaminated food lasting 3.5 h under humidified conditions at 37°C with agitation at 80 rpm which would have allowed for global expansion of bacteria present on lettuce. Thus, one viable *Salmonella* cell with an estimated doubling time of 20 min could be expected to yield 1,448 cells and lead to increased detectability. The incubation period was a compromise between ensuring that enough bacterial division cycles are achieved to facilitate detection versus a prolonged testing procedure that may not fit with the objective of delivering a test result before a suspect food is consumed under normal retail procedures in North America. Thus, longer incubation would probably be more advantageous to improve the changes of sensitive detection as previously demonstrated (Leonard et al., 2015), but may significantly lengthen the test procedure whereby a number of the initial steps in the protocol (e.g., stomacher, centrifugation) will have to be carried out after the first day of analysis. The second enrichment step is the global amplification of DNA which could result in hundreds to million-fold increase of the DNA available for library preparation prior to the sequencing run. A prolonged amplification procedure could be a rate-limiting step that might thwart the objective of delivering test results in a time frame that fits a precision and preventive food safety paradigm for perishable food. To identify the most appropriate fit-for-use procedure, we compared three different commercially available kits requiring approximately 4-, 8-, or 16-h incubation periods. We used a multiplex PCR to evaluate the presence of *Salmonella* DNA in each sample following amplification and found the GenomePlex kit to be the most sensitive, and fortunately it was also the shortest of the three protocols. The REPLI-g protocol was also quite sensitive, however it took twice as long, whereas the GenomiPhi protocol was in turn twice as long as the REPLI-g protocol; therefore, we adopted the GenomePlex kit. The average amplicon size of fragments reported for this kit was only 450 bp (manufacturers’ manual), but was optimal for the Ion Torrent sequencing platform wherein we used a template size of 400 bp. The GenomePlex procedure could, however, result in fragments as big as 2 Kbp or larger (data not shown) and was compatible with MinION long read sequencing technology with equivalent test sensitivity performance as the Ion Torrent sequencer even though the technology is primarily designed for analyzing very long DNA fragments of up to hundreds of Kb (manuscript under preparation). In line with developing a rapid assay, we also evaluated a much shorter ultra-rapid amplification procedure that could be completed in about 2 h but we found the method inadequate (data not shown). Our choice of amplification procedure appears to be global in nature without any evidence of preferential expansion of a subset of DNA over other subsets as shown by the ability of our test procedure to detect very low levels of *Salmonella* contamination as well as a diverse membership of the lettuce surface microbiota (see “Results” section under Bionformatic analysis of shotgun metagenomic sequencing reads from non-spiked and *Salmonella* spiked lettuce). Further work carried out in which other types of microbes including viruses, other bacteria and parasites were used for spiking equally led to a sensitive detection of the target by genome sequencing and bioinformatic analysis even when samples were spiked at low levels (data not shown). The combination of the sample preparation step that ensured minimal DNA of matrix origin was present, and two non-selective amplification steps facilitated the preponderance of DNA of pathogen of interest and the microbiota on the surface of lettuce. These sample preparation procedures are designed to facilitate the universal testing of any bacteria present such that the specific detection of a pathogen of interest, in this case *Salmonella*, is imposed on the bioinformatic procedure conducted at the end of the analysis and capable of identifying a single, specific sequence in the presence of a vast number of other sequences.

We employed the use of two Ion Torrent platform sequencers in this study, namely PGM and S5, both of which have identical template preparation procedures. To ensure a good coverage, each PGM chip was used to sequence a single sample, whereas two samples were sequenced on each S5 sequencer chip because of its higher capacity. The Ion Torrent platform server was programmed to automatically align the raw sequence reads from the spiked samples against the reference genome as a way of determining the presence of *Salmonella.* Thus, by the end of the run, and without initiating any external bioinformatic analysis, results indicating the proportion of reads that aligned with the reference genome were displayed, and this proved to be diagnostically accurate for all samples spiked with ≥10 cfu. Despite the ease of identifying a low-level, subclinical, dose of *Salmonella* contamination in spiked sample at the end of the sequencing run, we diligently explored bioinformatic procedures that could detect very low levels of *Salmonella* contamination. Three of the bioinformatic procedures are rapidly completed by directly analyzing unprocessed raw reads (fastq files), i.e., within 5 min. All analyses could be completed by an analyst without expert bioinformatics training by simply following a standard operating procedure that includes a how-to set of instructions. One of the procedures (CosmosID) relied on the use of a customized and well-curated database queried by raw sequences which is carried out by simply submitting the sequences to the server employing a user-friendly web portal. The fourth procedure, MEGAN analysis was the most involved, requiring an initial *de novo* assembly of raw reads followed by BLAST analysis of contigs and taxonomic designation using the MEGAN software, and could be completed in 60 min especially if a customized BLAST database is available.

All bioinformatics procedures used depended on the availability of specific markers. The three most rapid bioinformatics procedures are essentially a search for markers in our raw reads, two of which were based on well characterized markers for *Salmonella*, namely the *invA* gene (Galan and Curtiss, 1989) and a 523-bp Typhimurium-specific DNA fragment (Ogunremi et al., 2017b). The latter procedure, like all other sequenced based serotyping tools, is genome-marker dependent. For this reason, there is a need for the urgent identification and characterization of markers that can be used for genus-, species-, and serotype- and other subtype-specific characterization of foodborne pathogens. Many markers are already known, however, for the purpose of shotgun metagenomic sequencing where typically a fraction of the genome length is covered, detection procedures will benefit from the use of bioinformatic approaches that are based on a search for a large number of diagnostic markers. These markers could be genes, gene fragments, non-coding sequences, indels or even single nucleotide changes as shown by previous work (Laing et al., 2017). The CosmosID procedure which relies on k-mers and a proprietary database showed a superior performance in this study.

All samples including those contaminated with 1 – 9 cfu could be detected by performing all four bioinformatics procedures on ≥6 M raw reads within 2 h using a single computer (≥64 GB Random Access Memory). For those samples spiked with ≥9 cfu/25 g, analysis with either *invA* detection, 523-bp DNA fragment detection, MEGAN analysis or by CosmosID, was sufficient even when tested using the Ion Torrent PGM with its lower sequencing output. Our study allows us to characterize the strains by identifying serovar-specific markers (this study), antibiotic resistance genes, prophage sequences and clustering (unpublished observations). For food safety, it is important that low numbers of contaminants are detected given that they could result in an illness. In one instance, as few as 28 cfu per serving of ice cream was shown to have caused an outbreak of salmonellosis (Vought and Tatini, 1998).

WG-CIDT procedures are designed to overcome well known limitations of culture procedures, notably, delays in obtaining conclusive results which in some cases can stretch to 1–2 weeks, overgrowth of a pathogenic contaminant by the non-hazardous microbiota (false negative), low throughput and significant labor cost. Yet, culture procedures do have some distinct advantages including the definitive results when successful isolation has been completed on a positive sample. In addition, culture procedures have been developed to enable the amplification of the contaminant often through many steps of enrichment, which is the major contributor to the delay in obtaining test results. Due to the rapid analysis, a CIDT procedure will likely be adopted for routine use if it produces reliable test results that are comparable with the culture procedure, especially in terms of specificity.

Routine culture as a diagnostic procedure for detecting contaminated food is, however, not a sensitive procedure. Recovering low numbers of *Salmonella* in food (e.g., ≤100 cfu/25 g of food) is very difficult and requires careful and multiple enrichment procedures. Diagnostic laboratories have adopted the use of real time PCR (e.g., BAX analysis) to screen cultures during enrichment procedures so that a more rigorous *Salmonella* recovery effort can be focused on PCR positive samples. Thus, combination procedures are routinely used for *Salmonella* diagnostics which take time to complete, increase costs, and can create frustrations when results of multiple tests do not agree (e.g., positive PCR but failure to isolate). A recently described procedure using a combination of *Salmonella* targets indicated that PCR screening can now be carried out effectively early in the enrichment procedures (Nadin-Davis et al., 2019). In a similar fashion, culture procedures can still be carried out, perhaps more effectively, on samples shown to be positive by the culture-independent method if the recovery of an isolate is deemed important, especially for legal reasons.

The food recall system is an important element of the current public food safety system triggered often, but not exclusively, by episodes of human illnesses leading to the identification of a likely food source with the goal of curtailing further spread that would have been caused by the consumption of contaminated food^7^. This way, an ongoing outbreak could be interrupted by removing the source of infection. The epidemiological procedures and the laboratory testing required to link illnesses with a food source can collectively take weeks and, over this time period, the contaminated food is still available to consumers. Other constraining factors such as the need to conduct multiple tests on a pathogen (e.g., culture, phenotypic tests, subtyping, genome sequencing of isolates, etc.), which are typically carried out in different laboratories and could require shipping of samples, contribute to the delay. In addition, food types such as leafy greens and fruits with short shelf lives are typically consumed or discarded before an investigation is completed and a source may never be identified and robs investigators of knowledge that could be used for future control measures. Despite the above limitations, the diagnostic procedures currently in use have led to notable successes in breaking the transmission of foodborne illnesses especially those involving pathogens that persist in food and the food processing environment and in other protracted outbreaks. Nevertheless, the advent of newer and more powerful pathogen detection technologies allows the replacement or improvement of the old procedures with novel, innovative, proactive, precision-based and preventive systems where the goal is to avert illnesses altogether.

Another important limitation with current food safety diagnostic procedures is that the test to be carried out is determined *a priori* which nullifies the objective of a comprehensive assessment of the risk present in a food product. The Public Health Agency of Canada has estimated that 60% of foodborne illnesses, or 2.4 million cases (out of 4 million), are due to unspecified agents and this number included unculturable microbes (Thomas et al., 2015). Thus, a universal testing procedure that can lead to the detection of all possible contaminants will revolutionize food safety testing.

The application of genomics to CIDT is set to herald a new era of clinical microbiology in food safety, disease diagnoses and health of animals and humans (Forbes et al., 2017). While efforts to completely abandon the recovery and isolation of pathogens from the environment, and from animal and human hosts, are not altogether desirable, there is a tremendous gain to be realized in implementing WG-CIDT where action can be taken to reduce food risks and promptly safeguard animal and human health before the causal agents can be isolated in the laboratory. The universality afforded by a culture-independent genomics procedure will obviate the splitting of samples to carry out a specific enrichment procedure for each suspected organism. In some cases such as a suspected bioterrorist attack, there may not be any information about the type of organism suspected and a universal approach will remove most of the guess work, or at least reduce it considerably to specific searches for sequences that are already present in the raw reads.

There is a considerable interest and motivation to develop rapid methods for detecting *Salmonella* and other pathogens in food. Biosensor based procedures may well be the most rapid method available with claims of less than 1 h assay time to detect *Salmonella* and in one notable case only 2 min to detect *Escherichia coli* (Waswa et al., 2007) not counting the time taken to remove or extract bacteria from the food and concentrate into a small volume of sample buffer. Nucleic acid-based procedures involving variation of amplification procedures such as polymerase chain reaction or isothermal methods (Chen, 2008; Nam et al., 2005; Wu and Levin, 2015) which can be completed in up to 3 h of assay time are now available. These rapid assays tend to have relatively low sensitivity at >10^2^ cfu per ml (Zhao et al., 2014). Sensitivities are improved to 5 cfu per 25 g of food when the assays are done after a period of enrichment of up to 20 h (Kawasaki et al., 2005). To further improve test sensitivity, bacterial isolation using immunomagnetic beads targeted at *Salmonella* have been attempted (Leon-Velarde et al., 2009). Even when a very high sensitivity is attained, these tests provide information limited to genus level detection. For *Salmonella*, identification and serovar level characterization are of critical importance for food safety investigations and for this reason assays exploiting the microbial genome can be expected to display superior performance.

In this study, we employed a shotgun whole-genome metagenomics approach in contrast to the majority of bacterial metagenomic sequencing procedures that are based on the use of 16s rDNA kits. Ribosomal DNA can be an attractive target for both metagenomics and taxonomic analyses because the sequence occurs as repeats in bacteria leading to increased sensitivity. The combination of variable and conserved nucleotide sequences in different parts of the rDNA sequence provides a very attractive target for taxonomic analysis. Nevertheless, rDNA is a limited target compared to the entire genome and cannot be reliably used for subspecies level differentiation such as *Salmonella* serovar designation (Adeolu et al., 2016). In some bacteria organisms, its use for even species level designation is not as robust (Rajendhran and Gunasekaran, 2011). Thus, targeting the entire genome, or a considerable portion, as in shotgun whole-genome sequencing, especially when there are well-designated markers to probe the raw reads, will provide a more desirable outcome for identification of bacterial food contaminants and their characterization at the serotype/serovar level (Leonard et al., 2016).

Our CIDT detection method for *Salmonella* is apparently more sensitive than the one described for shiga-toxin *E. coli* by Leonard et al. (2015) which achieved a detection rate of 10 cfu per 100 g of spinach. Their approach required an 8 h selective enrichment for the target shiga-toxin producing *E. coli* and did not work as well if enrichment was not selective. We used a non-selective “double enrichment” procedure which included a number of cycles of multiplication of contaminants on the food surface and a global amplification of all extracted DNA. Other published procedures are able to achieve a detection limit of 10–70 cfu/g, i.e., 250–1,750/25 g (Pérez-Rodríguez et al., 2014; Rodriguez-Lazaro et al., 2014) which makes our assay more sensitive. Recently, Hyeon et al. (2018) combined selective immunomagnetic separation of *Salmonella* with a multiple displacement amplification procedure to prepare DNA template that were sequenced with the Illumina MiSeq sequencing procedure followed by extensive bioinformatic procedures including bacterial genome assembly. The procedure led to the identification of *Salmonella* in chicken and serotype designation of *Salmonella* Enteritidis except at low level contamination of a theoretical 2.5 cfu/25 g of chicken. Our assay has a higher sensitivity, avoids the cost and time required for immunomagnetic selection and lends itself to a universal application whereby any bacterial contaminant could conceivably be detected if present on fresh vegetables.

The time period from the first handling of contaminated lettuce to completing all bioinformatic analysis was 28 h and was shorter or similar to time reported by other genome-based assays (Leonard et al., 2016; Hyeon et al., 2018). We are currently employing the use of nanopore sequencing to accelerate the speed of testing and reduce assay period to at least half the time and to develop a mobile model that can be applied to unintentional and intentional contamination of food.

## Data Availability Statement

Raw metagenome reads have been deposited in GenBank under Bioproject PRJNA 593850 in the Sequence Read Archives (SRA).

## Author Contributions

DO conceived the idea. AD, SN, RG, KO, IM, HH, M-OD, LG, RL, NH, MD, BD, SM, and LM participated in the development of idea, protocol development, and data evaluation. AD, SN, RG, KO, IM, and DO developed the sample preparation protocol and optimization. AD, RG, KO, and IM carried out the genome sequencing. M-OD, NH, MD, LG, HH, RG, SM, and DO analyzed and evaluated the data. DO wrote the manuscript, and was assisted in the early stages by LM. All authors read, corrected, and approved the manuscript.

## Conflict of Interest

NH and MD are employed by CosmosID Inc.

The remaining authors declare that the research was conducted in the absence of any commercial or financial relationships that could be construed as a potential conflict of interest.

## Footnotes

1 https://www.canada.ca/en/public-health/services/laboratory-biosafety-biosecurity/pathogen-safety-data-sheets-risk-assessment.html

2 www.pall.com

3 http://www.lifetechnologies.com/ca/en/home/brands/ion-torrent.html

4 https://blast.ncbi.nlm.nih.gov/Blast.cgi

5 https://ab.inf.uni-tuebingen.de/

6 https://app.cosmosid.com/samples

7 http://www.inspection.gc.ca/about-the-cfia/newsroom/food-safety-system/food-recalls/eng/1332206599275/1332207914673
